# Holistic processing of faces and words predicts reading accuracy and speed in dyslexic readers

**DOI:** 10.1371/journal.pone.0259986

**Published:** 2021-12-15

**Authors:** Nuala Brady, Kate Darmody, Fiona N. Newell, Sarah M. Cooney

**Affiliations:** 1 Perception Lab, School of Psychology, University College Dublin, Belfield, Dublin, Ireland; 2 School of Psychology, Trinity College Dublin, Dublin, Ireland; Kobenhavns Universitet, DENMARK

## Abstract

We compared the performance of dyslexic and typical readers on two perceptual tasks, the Vanderbilt Holistic Face Processing Task and the Holistic Word Processing Task. Both yield a metric of holistic processing that captures the extent to which participants automatically attend to information that is spatially nearby but irrelevant to the task at hand. Our results show, for the first time, that holistic processing of faces is comparable in dyslexic and typical readers but that dyslexic readers show greater holistic processing of words. Remarkably, we show that these metrics predict the performance of dyslexic readers on a standardized reading task, with more holistic processing in *both* tasks associated with higher accuracy and speed. In contrast, a more holistic style on the words task predicts less accurate reading of both words and pseudowords for typical readers. We discuss how these findings may guide our conceptualization of the visual deficit in dyslexia.

## Introduction

Developmental dyslexia is characterised by difficulties in learning to read that are unexpected in light of a child’s cognitive abilities and educational opportunities [1]. These difficulties can persist into adulthood, and reading may be slower and new vocabulary challenging to college students with dyslexia [2, 3]. While differences in visuo-temporal processing are considered integral to dyslexia by many [4, 5], research on dyslexia has focused predominantly on phonological processing, with reported impairments in phonological coding, rapid naming and verbal short-term memory [6].

The view that visual problems in dyslexia are secondary to a ‘core phonological deficit’ [7] endures, in part, because it resonates with the dual-route theory of reading [8]. By this account, learning to read involves the acquisition of distinct phonological and orthographic skills. Phonological coding, crucial in early reading, establishes a mapping between letters and their associated sounds. Orthographic coding refers to the representation of the visual form of words–including groupings of letters that signal spelling regularities–and, in time, enables word recognition without the need to access phonological information at the pre-lexical level [9]. This *lexical* route to word sounds is assumed to underlie fluency. It has been proposed that poor phonological processing in dyslexia may hinder the development of spelling-sound mappings, thus preventing children from learning precise orthographic information about words, and ultimately from attaining fluency [10].

This emphasis on the primacy of phonological deficits in dyslexia has been challenged in recent years. For example, while the account has much appeal for languages with opaque orthographies such as English—where spelling-sound correspondences are particularly obtuse [11] - dyslexia is also common in languages with more transparent orthographies. Using a cluster analysis of WISC-IV data from over 300 Italian children with dyslexia [12] report two distinct groups, both with impairment in visual processing, but with only one group having additional impairment in phonological processing. The argument for a more direct role of visual impairment in dyslexia, specifically in visual attention, is also made by Valdois and colleagues [13]. [14] show deficits in visual attention span in large samples of English and French dyslexic children, independent of impairment in phonological processing. Such findings argue for a reconceptualization of dyslexia as a multifaceted disorder, one in which anomalous visual processing may occur independently of or in conjunction with poor phonological processing. However, the nature of the visual deficit in dyslexia is still poorly understood. Here we investigate whether ‘holistic processing’, defined as obligatory attention to all parts of a stimulus is different in dyslexia.

Most pertinent to the research we present in this paper are a number of recent studies reporting subtle deficits in visual cognition in dyslexia which suggest that anomalous visual processing is less specific to words than previously considered. These studies were inspired in part by reports of hypoactivation in left fusiform gyrus in both adults and children with dyslexia [15–17]. This region of the brain includes the visual word form area (VWFA) which responds preferentially, but not exclusively, to printed words [18] and which is adjacent to regions that respond preferentially to faces. Therefore, deficits in both word and face recognition, reflecting a general impairment in ventral stream processing, might occur in dyslexia [19, 20].

Sigurdardottir et al. [20] investigated whether dyslexic and typical readers differ in their face and object recognition abilities. Nineteen self-reported dyslexic and controls were tested on the Cambridge Face Memory Test (CFMT), the Vanderbilt Holistic Face Processing Test (VHFPT), and the Vanderbilt Expertise Test (VET). Dyslexics showed poorer memory for faces in the CFMT, being less accurate whether the task was performed with upright or inverted faces. As face recognition was comparably compromised across groups when the faces were inverted—a manipulation thought to induce a switch from holistic to part-based processing—this suggests that the poorer performance of dyslexics does not reflect a specific impairment to holistic processing of faces. Similarly, in the VHFPT dyslexics were less accurate overall than controls. Finally, as dyslexic readers were less accurate than controls on the VET but not on a control colour recognition task, this also suggests that hypoactivation in left fusiform gyrus may result in subtle impairments in within-category object discrimination.

Employing a number of challenging perceptual tasks, [19] found that dyslexics were slower than typical readers in matching faces across different viewpoints, but that the groups were similarly hindered when matching between upright target faces and inverted test faces, suggesting that holistic processing of faces is not specifically impaired. Similarly, dyslexics were less accurate in discriminating pairs of morphed images of faces but not in discriminating pairs of morphed images of cars. Finally, [21] asked participants to match images of 3D modelled faces and novel object. Briefly, in Exp 1 accuracy in the face matching task predicted reading problems in a sample of university students, although not distinguishing within groups of competent readers or within groups of poor readers. In Exp 2, performance in the novel objects matching task did not predict whether participants were dyslexic or typical readers, but performance in the face matching task did. The authors conclude that visual problems in developmental dyslexia are specific to high level tasks involving words and faces with which people have extensive experience or expertise.

In this paper we ask whether ‘holistic processing’, a form of visual processing which is considered a hallmark of perceptual expertise by some [22, 23] is anomalous in dyslexia. Specifically, and for the first time, we compare holistic processing of words *and* of faces in participants with dyslexia and age-matched controls and we show that holistic processing of both faces and words predicts reading performance in the dyslexic but not in the typical reader group.

Holistic or configural processing has been proposed to underlie both face [24] and word [25] recognition. In the case of faces, it is generally agreed that an accurate representation of second-order facial configuration—the precise geometric arrangement of features in the face—underlies expertise in recognition [26]. Although often used synonymously with ‘configural processing’, the term ‘holistic processing’ is often reserved to describe the automatic processing of facial features as a perceptual whole or gestalt which makes individuation of features difficult and *it is in this sense* that we use the term in this paper. This automatic processing of facial features as a perceptual whole is illustrated by the *composite face illusion* whereby a single face, made by aligning images of the top and bottom half faces of different individuals, is perceived as a single facial identity [27, 28]. Even when directed to ignore one half of the composite, participants typically fail to selectively attend and some form of perceptual integration occurs. As expected, the composite face effect is considerably reduced when the two half faces are misaligned. The composite paradigm has recently been extended to the study of word recognition by [25] who show that expert readers are unable to ignore one part of a word when asked to attend to the other part of that word in a matching task. This suggests that holistic processing is not specific to face perception, but instead may occur as a result of repeated exposure or visual expertise with objects.

The current study explores whether ‘holistic processing’–as measured for both faces and words using comparable tests of performance—is anomalous in adults with dyslexia. For faces, we use the VHFP Test [29], a modern variant of the face composite test that dispenses with the alignment condition and focuses exclusively on the primary effect of congruency of the aligned faces. For words we use Wong’s Holistic Word Processing Task [25] which is based directly on the original face composite test and involves matching words under conditions which vary in congruency and alignment as described below. As in [25] Study 1, we define the congruency effect as the difference in performance between congruent and incongruent trials in the aligned condition, which matches the metric of [29]. These two measures of holistic performance are then used as predictors of participants’ scores on a standardized reading test.

## Methods

### Participants

Of 62 participants who took part in the study, data from three were excluded; one’s data were missing a very high proportion of trials (over 30%) and two had very high error rates coupled with very fast RT’s or alternating yes/no responses suggesting that the participants did not engage seriously with the task. Analyses were conducted on the final sample of 59 adults, 30 students with a formal diagnosis of dyslexia (17 female) and 29 students (19 female) who served as controls. Power analysis, using PANGEA [30] indicated that a sample size of 30 per group (Dyslexic/Typical Readers) would provide 98% power to detect a medium effect size (d = 0.45) for a two-way Group*Congruency ANOVA design. Participants were recruited from both University College Dublin and Trinity College Dublin, the students with dyslexia being registered with disability support services at their university which requires a formal diagnosis of dyslexia to be provided by a clinical or educational psychologist. Of the 30 students with dyslexia, one completed the words task only and one completed the faces task only due to time constraints.

The dyslexic and typical readers participants had a mean age of 25.0 years (SD = 8.1) and 25.86 years (SD = 11.0) and a t-test revealed no significant difference in age between the groups, *t*(57) = 0.35, *p* = .73. All participants self-reported normal or corrected to normal vision. While all participants reported ‘normal’ or ‘corrected to normal’ visual acuity for the purpose of the study, there were more reports of corrected vision and of other issues with vision among dyslexic participants as documented in Table 1. Using a binary classification of ‘normal vision’ and ‘other’, Pearson’s Chi-squared test showed *X*^*2*^ = 6.13, df = 1, *p* = 0.01.

**Table 1:** Vision by self-report.

Visual Issues	Control	Dyslexic	Sum
**Normal Vision**	18	8	26
**Short-sighted, corrected**	5	14	19
**Long-sighted, corrected**	5	2	7
**Use glasses for both TV & reading**	0	2	2
**Short-sighted, double vision from age 6**	0	1	1
**Long-sighted (corrected), plus lazy eye**	1	0	1
**Astigmatism**	0	1	1
**~5% vision in right eye**	0	1	1
**Monochromatic vision**	0	1	1
**Total**	29	30	59

*Note*. Participants were asked to report any issues with their vision.

The study was approved by the UCD and TCD Research Ethics Committee; in accordance with the Declaration of Helsinki all participants gave written, informed consent and were advised of their right to withdraw from the study at any time without prejudice.

### Materials and procedure

#### Reading tests

All participants completed two subscales of the Wechsler Individual Achievement Test (3rd Edition), the Word Reading and the Pseudoword Decoding tests. In the Word Reading test, participants were asked to read aloud 74 words from a test sheet and the number of words read at 30 seconds was noted as a measure of reading speed. Words read fluently were awarded 1 point and words pronounced incorrectly were awarded 0 points. The test was discontinued if the participant read 4 consecutive words incorrectly and participants were given a further opportunity to read any incorrectly pronounced words at the end of the session. The same procedure was followed for the Pseudoword Decoding test using a test sheet of 52 pseudo-words. All participants completed the reading tests first, after which the order of the faces and words tests was randomised across participants.

#### Vanderbilt Holistic Face Processing Task (VHFPT)

We used the VHFPT 2.0 version of the Vanderbilt Holistic Face Processing Test described and tested in [29]. As reported by the authors, the VHFPT 2.0 shows superior psychometric properties relative to prior holistic face processing measures, with higher internal consistency (0.56) than the composite task and with test–retest reliability of 0.49 (R = 0.94) after a 6 month delay. It produces large average effect size for holistic processing (η2p = 0.75) and is normally distributed in an adult population [29]. The stimuli, with order counterbalanced so that half the participants completed the words tasks first and the other half completed the faces task first, were presented on a 22-inch colour monitor (1280 x 1024 resolution) using a Dell PC running Presentation® software. Viewing distance was ~50cm.

The test utilizes grayscale images of composite faces, made by combining images from two individuals’ faces from a set of 360 unfamiliar Caucasian faces. The 3-alternative force choice (3AFT) task involves looking at a target region of a study face, while ignoring the rest of the face, and locating the matching identity in the same target region of one of three test faces, where one is the correct test face, and the two others are foils. There were nine target segment conditions: bottom two thirds (BTT); top two thirds (TTT); bottom third (BT); top third (TT); bottom half (BH); top half (TH); eyes; mouth; nose. There were 20 trials (10 congruent, 10 incongruent) per target segment and 60 trials (30 congruent, 30 incongruent) per face size (small, medium and large) as described by for a total of 180 trials. The target segment of the study face and the target segment of the (correct) test face were taken from two different images of the same person on both congruent and incongruent trials. On *congruent trials* the distractor segment of the correct test face was also matched in identity to the distractor segment of the study face. However, on *incongruent trials*, the distractor segment of the (correct) test face was not matched in identity to the distractor region of the study face. Specifically, the target region in both the study face and the (correct) test faces are from Person A. On the congruent trial, the non-target region of both the study and (correct) test face are from Person B. However, in the incongruent trial, while the target regions are matched in identity (Person A), the non-target regions of the study face and the (correct) test face are from two different identities (Persons A and C). See S1 Fig for graphical details.

On each trial, a study face which was a composite image of two different face images was presented for 2000ms with the target region of the face delineated by a red box. Participants were instructed to only focus on the target region and to ignore the rest of the face. A blank screen followed for 1000ms. Three test faces were then displayed, positioned horizontally, left, centre and right, until the participant made a response to indicate which one had the matching target region. Each of the three test faces were marked on the target region with a red box. Only one of the test faces contained the correct target segment identity (correct face) the two other test composites were incorrect foils. Participants were required to indicate which of the test faces contained the target segment of the study face by pressing one of three response keys on the keyboard, left image, centre image, right image, using keys F, G, & H. The experiment was preceded by three practise trials using composites created from Muppet faces that were presented in colour.

#### Word recognition task

The stimuli were identical to those used by [25] and were given freely by the first author for use in this study. The stimuli consisted of four-letter words created from ten sets (40 words in total). Each set was made up of four words from which the left and right halves could be alternated, e.g., as shown in S2 Fig left halves ‘br’ and ‘sl’ can be combined with right halves ‘im’ and ‘ow’ to create four distinct words, ‘brim’, ‘brow’, ‘slow’ and ‘slim’. Four test conditions were created. On congruent trials the study and test stimuli were entirely the same or different. On incongruent trials half of the study and test stimuli were the same and half were different. Each word was presented as both test and study stimulus equally in each of the four conditions. Half of the trials were presented in aligned conditions and half were presented misaligned in which the non-cued half of the word was moved approximately 1.7° vertically.

On each trial a fixation was presented for 500ms, followed by a study word for 400ms. This was replaced by a mask for 500ms, after which a cue appeared to the left or to the right of the mask for a further 300ms to indicate the target half of the study word. The test word, also cued on the same side, followed for 1500ms after which the screen went blank until the participant responded. Participants were required to indicate if the cued half of the test stimulus was the same or different as the same half of the study stimulus by pressing either “same” or “different” keys on a Cedrus RB-844 response box. Following [25] the study contained a total of 640 trials, with 16 blocks of 40 trials. Presentation of alignment conditions was counterbalanced, so that half the participants completed the aligned condition first and the other half completed the misaligned task first. All other conditions were randomized across participants. Participants completed 20 practice trials in advance of the experiment.

## Results

Data were analysed in R [31]. Welsh’s t-test is used by default for between-group comparisons and corrected degrees of freedom reported [32]. Effect sizes (Cohen’s *d)* are interpreted as originally suggested with *d* = 0.2, 0.5, 0.8 as small, medium and large effect sizes. For ANOVA, Greenhouse-Geisser corrections are used when Mauchly’s Test for Sphericity was significant and effect sizes are given by partial eta squared (*η*^*2*^_*p*_). We follow a conservative approach to removal of RT ‘outliers’ using exploratory data analyses (box- and-whisker and qq-normal plots) to note RTs which are obviously too fast (anticipatory errors) or much too slow (so there is a noticeable break in the upper extremes of the data) suggesting that the participant was not attending properly on the trial. Other methods, such as removing any RT above a fixed number of SDs above the mean for each person can be very problematic (despite their regular use as heuristics), as RT data are generally asymmetric with long right-tailed skew [33–35] so that distinguishing outliers from genuine high RTs is problematic.

### Reading tests

**Fig 1:**
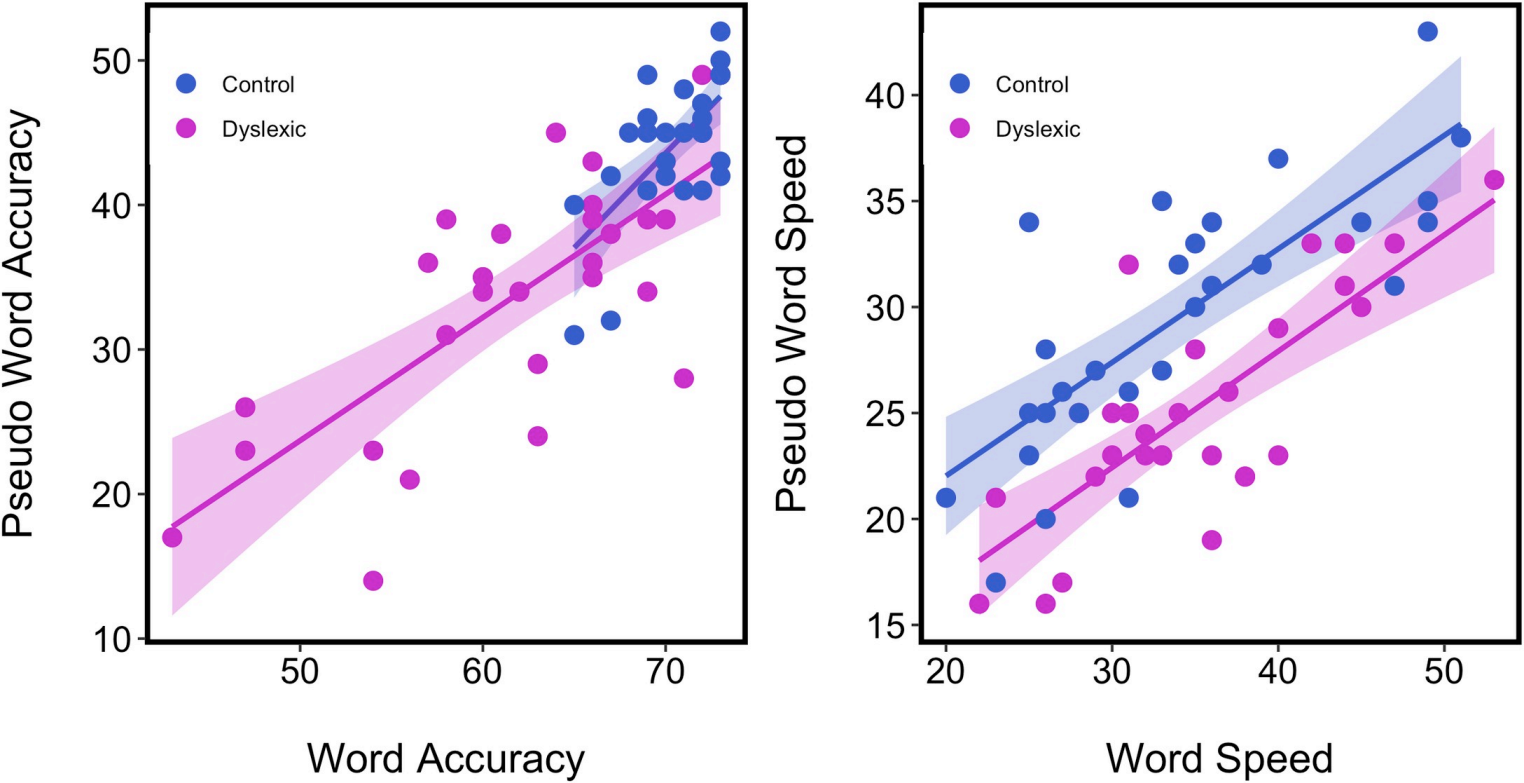
Pseudo-word accuracy and pseudo-word speed is plotted against word accuracy and word speed for both dyslexic and control participants. The shaded areas show the standard error bounds. Accuracy is reported as the number (out of 74) read accurately, Speed is the number read accurately within 30secs so that lower speed corresponds to poorer performance.

Fig 1 plots pseudo-word accuracy against word accuracy and pseudo-word reading speed against word reading speed for both dyslexic and control participants.

Accuracy scores for pseudo-words and words were highly correlated for both dyslexic, *r* = 0.74, df = 28, *p* < 0.0001, 95% CI [0.51, 0.87] and control, *r* = 0.67, df = 27, *p* < 0.0001, 95% CI [0.40, 0.83], groups, and for the combined groups, *r* = 0.82, df = 57, *p* < 0.0001, 95% CI [0.71, 0.89]. Similarly, speed scores for pseudo-words and words were highly correlated, for both dyslexic, *r* = 0.77, df = 28, *p* < 0.0001, 95% CI [0.57, 0.88], and control, *r* = 0.78, df = 27, *p* < 0.0001, 95% CI [0.58, 0.89], groups and for combined groups, *r* = 0.70, df = 57, *p* < 0.0001, 95% CI [0.54, 0.81]. While accuracy clearly discriminates the dyslexic and control groups for both words and pseudowords as shown in the Fig 1, the groups perform comparably with respect to word speed but not with respect to pseudoword speed where dyslexic students are slower (see also Table 2). Paired sample t-tests showed significant differences between groups in both word accuracy, *t*(34.87) = 5.89, *p* < 0.0001, *d* = 1.51, and pseudo-word accuracy, *t*(44.57) = 5.71, *p* < 0.0001, *d* = 1.47. The difference between groups in word reading speed was not significant, *t*(54.06) = -0.36, *p* = 0.71, *d* = -0.09, whereas for pseudo-word reading speed the control group were faster, *t*(54.96) = 3.05, *p* = 0.004, *d* = 0.79. Summary statistics are provided in Table 2.

**Table 2:** Scores for WIAT word and pseudo word reading test.

Group	Word Accuracy	Pseudoword Accuracy	Word Speed	Pseudoword Speed
**Dyslexic**	61.93 [59.09, 64.78]	33.87 [30.58, 37.15]	34.87 [32.15,37.58]	25.10[23.17,27.03]
**Control**	70.55 [69.64, 71.46]	44.31 [42.53, 46.09]	34.10 [30.72, 37.48]	29.59 [27.27, 31.89]

*Note*. Means and 95% CI. Word and Pseudoword Accuracy are the number (out of 74) read accurately, Word and Pseudoword Speed are the number read accurately within 30secs.

### Faces test

#### Accuracy & response time

**Fig 2:**
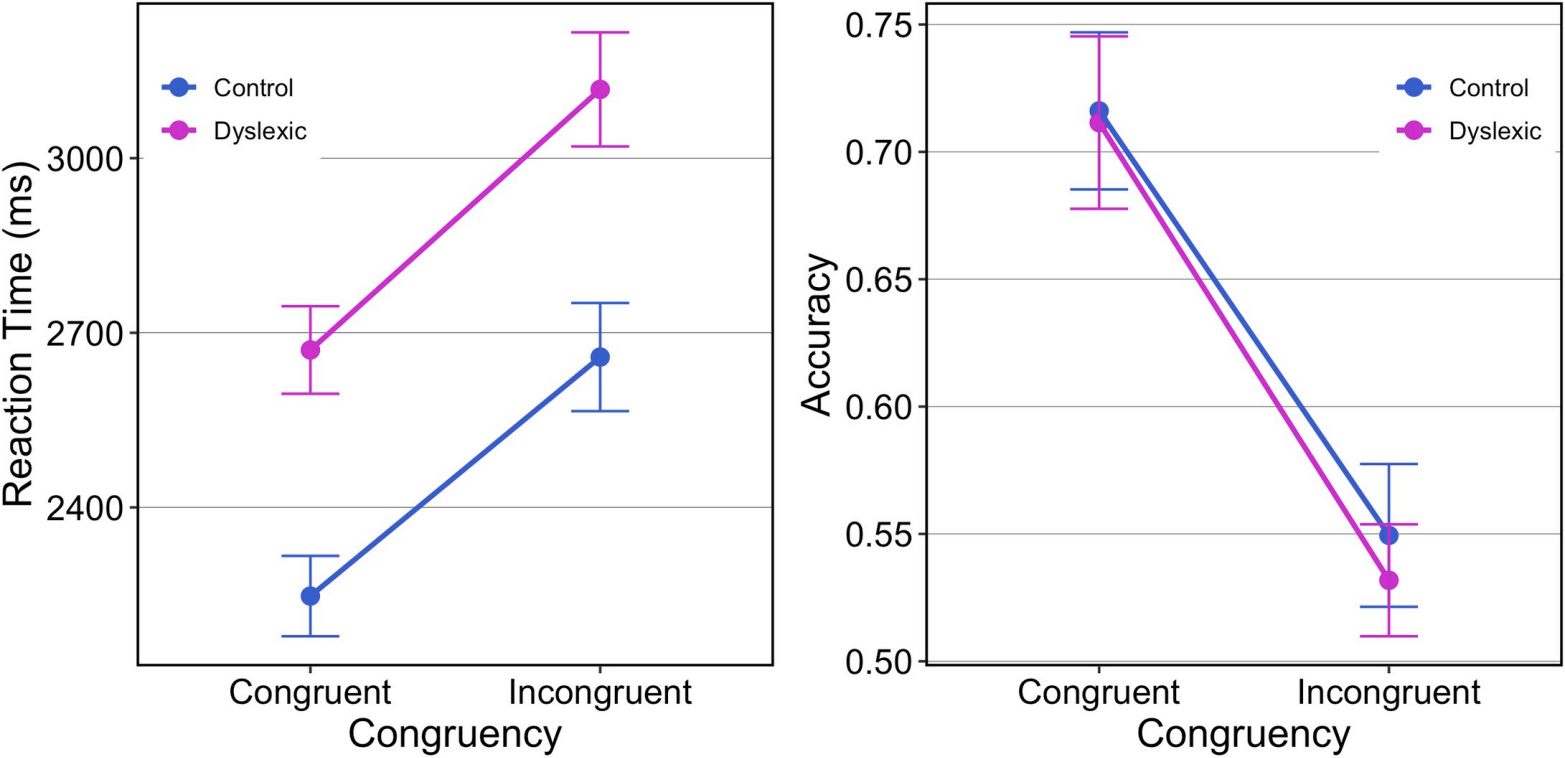
**RT (left) and accuracy (right) are plotted by congruency and by group in the faces task.** Error bars show 95%CI about the mean. Accuracy is expressed as a proportion (0.0 to 1.0) and chance performance in this task is 0.33.

The overall error rate was comparable for control (36.7%) and dyslexic (37.8%) groups and in keeping with the high rates reported by [29] who explain that the task is purposively challenging. Exploratory data analyses highlighted a small number of RTs less than 400ms or greater than 20000ms (less than 0.05% of all trials) that were removed as outliers. Fig 2 plots accuracy by group and congruency which suggests an effect of congruency only. In contrast, although this was not an explicit reaction timed task, the plot of RT on correct trials suggests an effect of congruency and group.

For accuracy, mixed-effects ANOVA with a within-subjects factor of *Congruency* and a between-subjects factor of *Group* yielded main effects of *Congruency*, *F*_(1, 56)_ = 231.38, *p* < .0001, $\eta_{p}^{2}$ = .81, where accuracy is higher for congruent [*M* = 71.4%, *SD* = 8.4%] than incongruent [*M* = 54.0%, *SD* = 6.6%] trials. Neither the main effect of group, *F*_(1, 56)_ = 0.45, *p* = .50, $\eta_{p}^{2}$ = .008, nor the Congruency*Group interaction, *F*_(1, 56)_ = 0.33, *p* = .57, $\eta_{p}^{2}$ = .006, were significant. For RT, the main effects of *Group*, *F*_(1, 56)_ = 3.83, *p* = .055, $\eta_{p}^{2}$ = .06, and of *Congruency*, *F*_(1, 56)_ = 104.35, *p* < .0001, $\eta_{p}^{2}$ = .65, are of note while the Congruency*Group interaction, *F*_(1, 56)_ = 0.67, *p* = .41, $\eta_{p}^{2}$ = .01, was not significant. With respect to group, controls were faster, M = 2456ms, 95% CI [2218, 2698], than dyslexics, M = 2892ms, 95% CI [2669, 3116]. At the request of a reviewer, this analysis was repeated using median RT and showed a significant main effects of *Group*, *F*_(1, 56)_ = 4.61, *p* = .036, $\eta_{p}^{2}$ = .08, and of *Congruency*, *F*_(1, 56)_ = 66.72, *p* < .0001, $\eta_{p}^{2}$ = .54, while the Congruency*Group interaction, *F*_(1, 56)_ = 1.34, *p* = .25, $\eta_{p}^{2}$ = .02, was not significant.

### Words test

#### Response time & sensitivity

**Fig 3:**
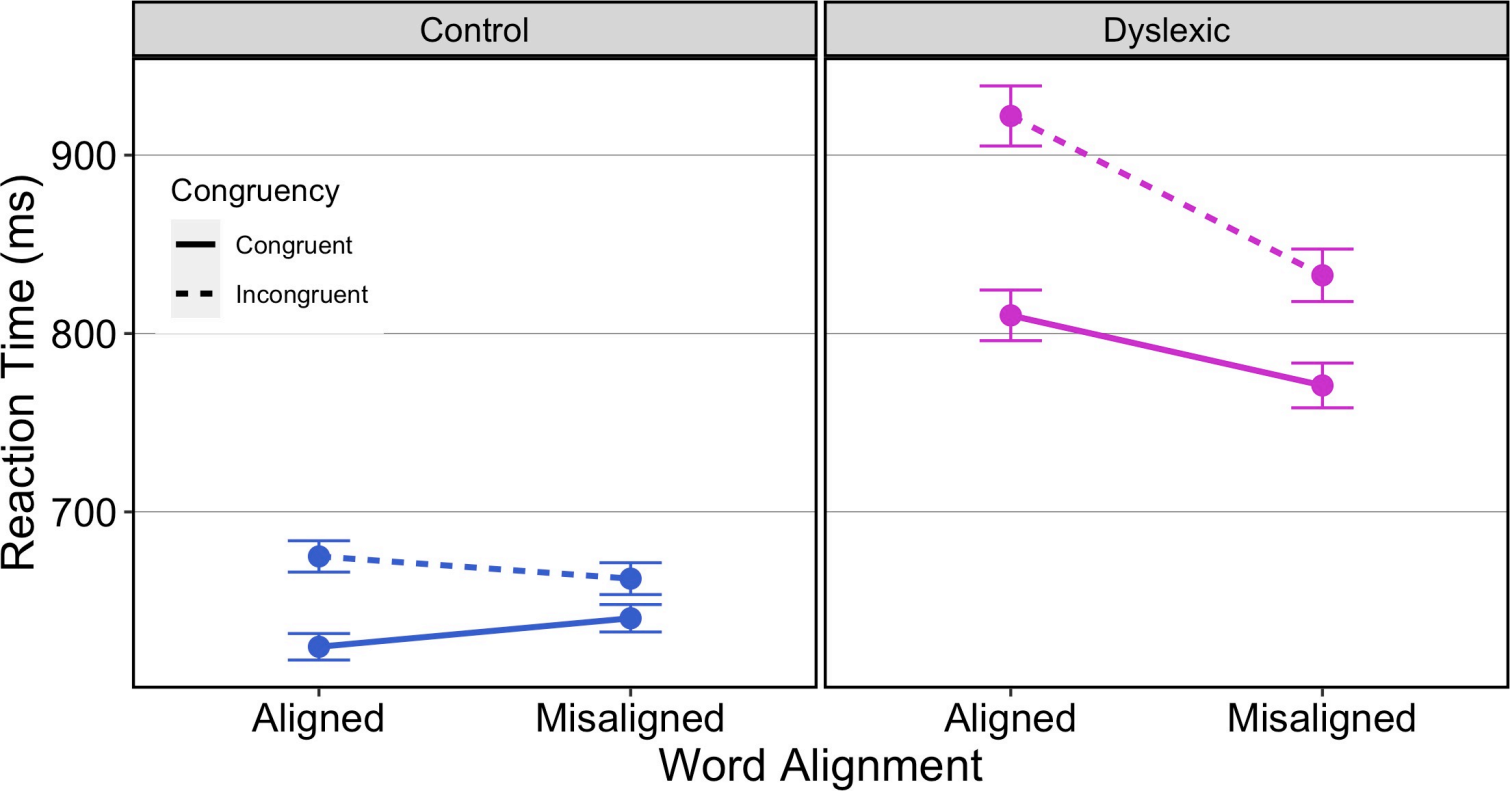
RT for correct trials is plotted in separate panels for control and dyslexic participants, with condition of alignment indicated on the x-axes and condition of congruency indicated by line type. Error bars 95%CI about the mean.

Exploratory data analyses highlighted a small number of RTs less than 200ms or greater than 8000ms (less than 0.04% of all trials) that were removed as outliers. Overall, errors were made on 5.34% of trials, 2.83% for dyslexic and 2.51% for typical readers. Fig 3 plots RT on correct trials by group and by conditions. Dyslexic participants are slower than controls overall, and both groups are slower in the incongruent than in the congruent condition.

Mixed effects ANOVA showed a significant main effect of *Group*, *F*_(1, 56)_ = 10.91, *p* = .002, $\eta_{p}^{2}$ = .16, with dyslexics, M = 832.8ms [SD = 495.5ms], slower than controls, M = 650.4ms [SD = 278.7ms]. There was a significant main effect of *Congruency*, *F*_(1, 56)_ = 55.22, *p* < .0001, $\eta_{p}^{2}$ = .50, with slower performance on incongruent, M = 771.9ms [SD = 378.9ms], than on congruent trials, M = 711.5ms [SD = 378.9ms]. And, as expected from [25] there was a significant Congruency * Alignment interaction, *F*_(1,56)_ = 20.78, *p <* .0001, $\eta_{p}^{2}$ = .27, the effect of congruency being greater in aligned than misaligned trials. Additionally, the Group * Congruency interaction, *F*_(1,56)_ = 9.76, *p* = .003, $\eta_{p}^{2}$ = .15, was significant and is explored further below in the *Congruency Effect* section. The three way Group*Alignment*Congruency interaction was not significant at conventional levels, *F*_(1,56)_ = 1.69, *p* = .197, $\eta_{p}^{2}$ = .03. Using median RTs, as above for the faces task, the analysis showed a significant main effect of *Group*, *F*_(1, 56)_ = 11.53, *p* = .001, $\eta_{p}^{2}$ = .17, and of *Congruency*, *F*_(1, 56)_ = 38.25, *p* < .0001, $\eta_{p}^{2}$ = .41. There was a significant Congruency * Alignment interaction, *F*_(1,56)_ = 4.12, *p =* .047, $\eta_{p}^{2}$ = .07, and a significant Group * Congruency interaction, *F*_(1,56)_ = 9.01, *p* = .004, $\eta_{p}^{2}$ = .14. The three way Group*Alignment*Congruency reached significance at conventional levels, *F*_(1,56)_ = 7.19, *p* = .02, $\eta_{p}^{2}$ = .11. For dyslexics, the Alignment*Congruency effect was significant, *F*_(1,28)_ = 21.17, *p* < .0001, $\eta_{p}^{2}$ = .43, with a more marked difference in RT between congruent and incongruent trials in the aligned that in the misaligned condition. And for typical readers, the Alignment*Congruency effect was significant, *F*_(1,28)_ = 8.35, *p* = .007, $\eta_{p}^{2}$ = .23, with a more marked difference in RT between congruent and incongruent trials in the aligned that in the misaligned condition.

D-prime (*d’)*, a measure of sensitivity that is independent of response bias [36] was calculated. Mixed effects ANOVA revealed no significant main effect of *Group*, *F*_(1, 56)_ = 0.34, *p* = .561, $\eta_{p}^{2}$ = .006, a significant main effect of *Congruence*, *F*_(1, 56)_ = 74.26, *p* < .0001, $\eta_{p}^{2}$ = .57, with higher sensitivity on congruent than incongruent trials. The Congruence * Group interaction was significant, *F*_(1,56)_ = 4.87, *p* = .03, $\eta_{p}^{2}$ = .08, as was the Congruence * Alignment interaction, *F*_(1,56)_ = 30.05, *p* < .0001, $\eta_{p}^{2}$ = .35. Regarding the former, planned comparisons show an effect of *Congruency* for both dyslexic, *F*_(1,28)_ = 54.63, *p* < .0001, $\eta_{p}^{2}$ = .66, and controls, *F*_(1,28)_ = 22.15, *p* < .0001, $\eta_{p}^{2}$ = .44, that is more marked for dyslexic participants. Regarding the latter, planned comparisons show an effect of *Congruency* in aligned (*p* < .0001) and misaligned (*p* = .0003) conditions, that is more marked in the aligned condition.

### Congruency effect

#### Faces & words

**Fig 4:**
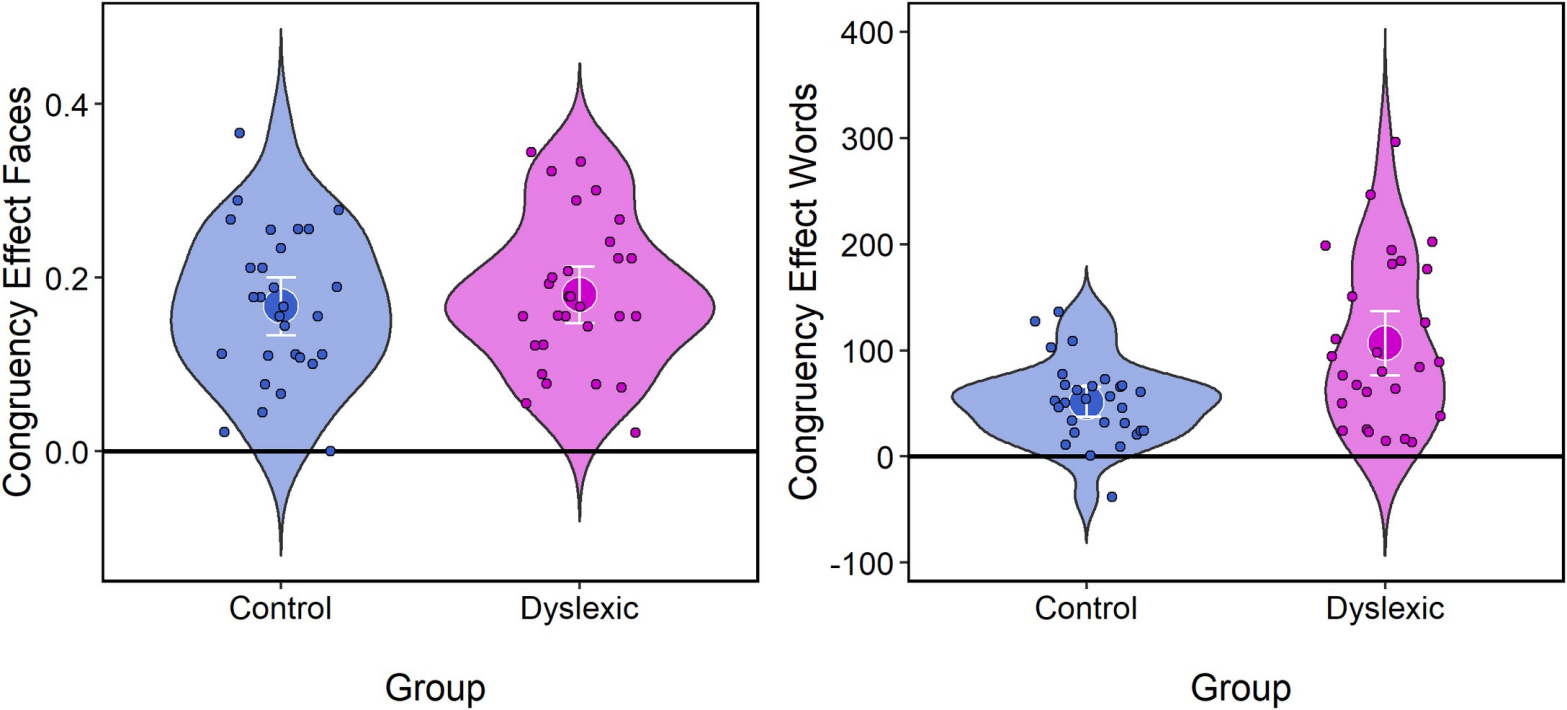
Congruency effect on the faces (left) and words on aligned trials (right) task for both participant groups. The congruency effect for faces is defined with respect to accuracy and the congruency effect for words with respect to RT, see text for details. The violin plots include individual subject points and show 95% CI about the mean.

The *congruency effect* in the faces task is defined as the difference in accuracy on incongruent and congruent trials, and serves as a metric of ‘holistic processing’, operationalized in terms of obligatory attention to all parts of the face [29]. Participants who can attend solely to the highlighted region of the face should perform with comparable accuracy on congruent and incongruent trials and will have a low *congruency effect*. In contrast, those with a more holistic style of processing will be more easily distracted by information from the irrelevant or ‘to be ignored’ face region and so be less accurate on incongruent than on congruent trials leading to a higher *congruency effect*. By similar logic, the *congruency effect* in the words task is defined as the difference in RT between incongruent and congruent trials on aligned trials and serves as an index of how much the irrelevant information interferes with observers’ judgments [25]. Fig 4 plots the congruency effect in the faces (left) and in the words (right) task for both dyslexic and typical readers, with. In both tasks, both groups show evidence of holistic processing, of comparable magnitude in the faces tasks but with dyslexic participants showing a stronger effect than controls in the words task. One participant from the dyslexic group was removed as their congruency effect on the words task was over four standard deviations from the group mean in the positive direction, i.e., they showed an extremely high congruency effect. They are not represented in Fig 4 nor in the analyses below.

In the faces task, ANOVA showed no effect of *Group*, *F*_(1, 56)_ = .34, *p* = .56, $\eta_{p}^{2}$ = .006, the congruency effect being comparable for dyslexic [*M* = .18, *SD* = .09] and control [*M* = .17, *SD* = .09] participants. However, in the word task, ANOVA there was a significant effect of *Group*, *F*_(1, 55)_ = 11.90, *p* = .001, $\eta_{p}^{2}$ = .18, the congruency effect being greater for dyslexic [*M* = 107.0, *SD* = 77.3] than controls [*M* = 51.4, *SD* = 37.5] participants. Although not pertinent to the analysis below, where the focus is on the congruency effect on aligned trials, we note that a further mixed effects ANOVA shows a significant effect of both *Group*, *F*_(1,55)_ = 10.38, *p* = .002, $\eta_{p}^{2}$ = .17, and of *Alignment*, *F*_(1,55)_ = 19.52, *p* < .0001, $\eta_{p}^{2}$ = .26. The Group* Alignment interaction was not significant (*p* = .28). This shows that both dyslexic and control participants were susceptible to interference from the unattended part of the stimuli, and more so when the two halves of words were properly aligned.

### Congruency effect as predictor of reading scores

**Fig 5:**
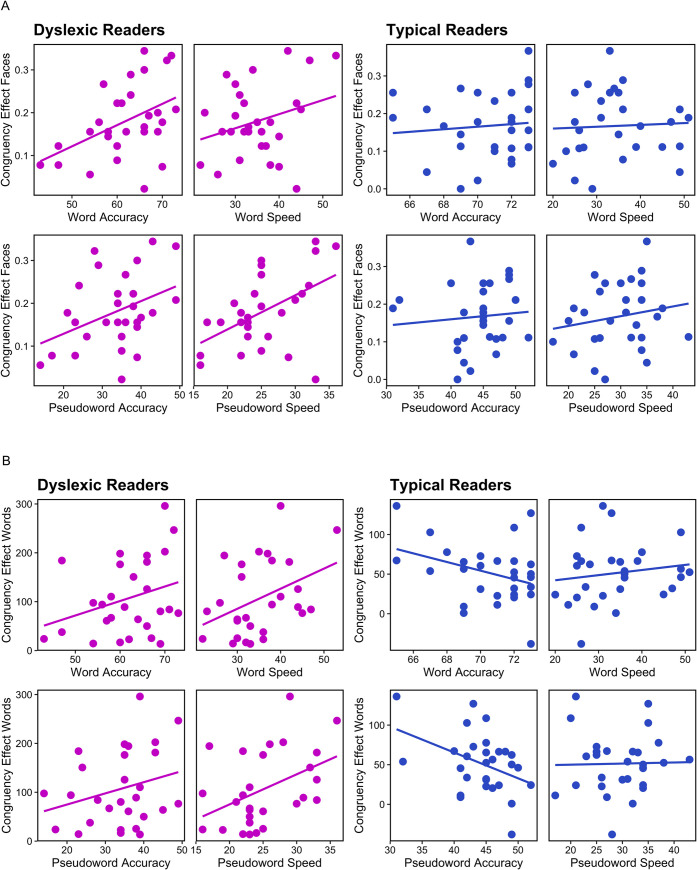
Scatter plots of the congruency effect in the faces task (A) and the words task (B) by each of the four WIAT reading score metrics with separate plots for dyslexic (magenta) and typical (blue) readers. Accuracy is reported as the number (out of 74) read accurately, Speed is the number read accurately within 30secs so that lower speed corresponds to poorer performance.

**Fig 6:**
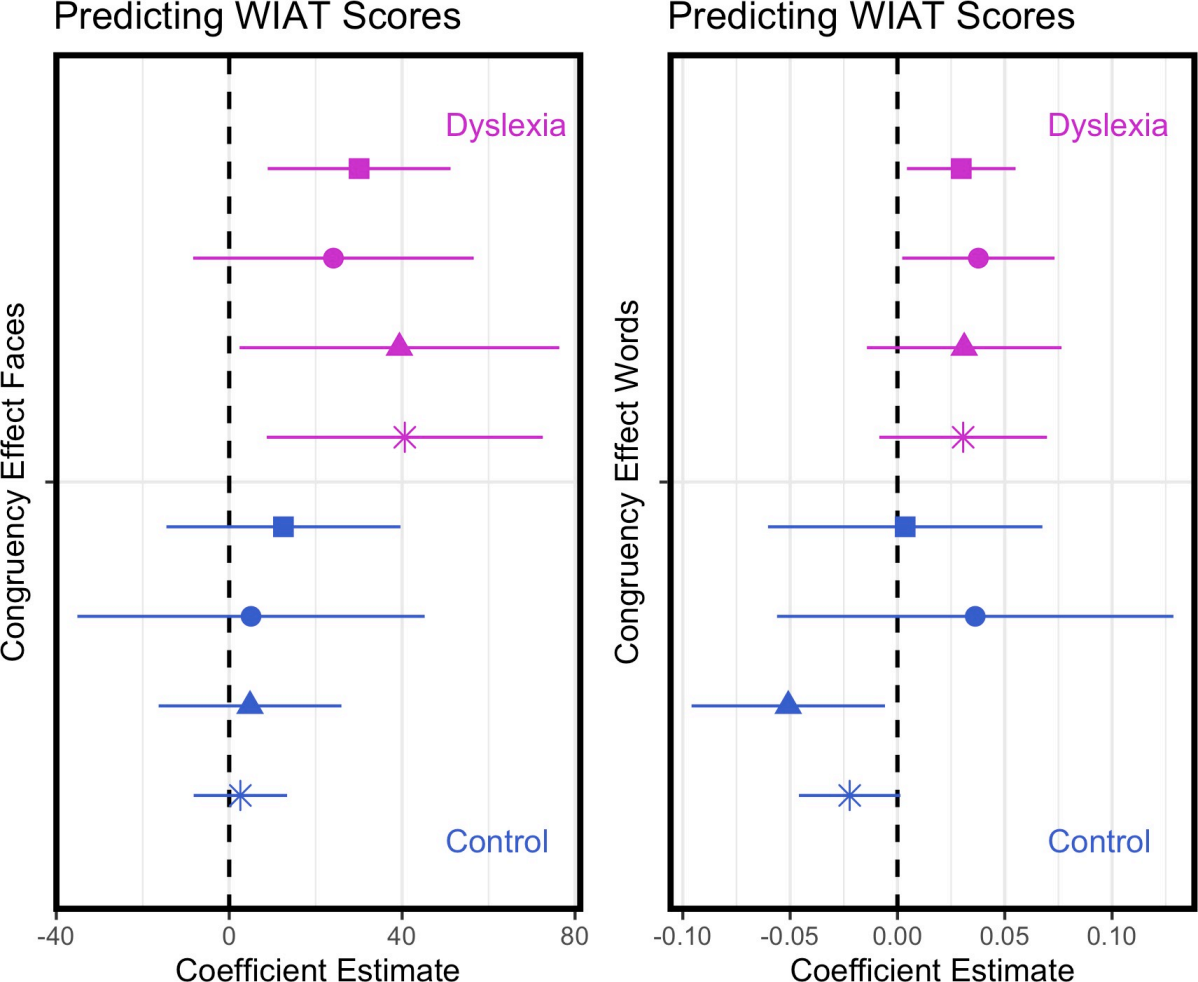
**Dot-and-whisker plots showing the slope coefficients (with 95% CIs) from linear models regressing reading score metrics on the congruency effect for faces (left) and for words (right).** Symbols key: Word Accuracy (square), Pseudoword Accuracy (circle), Word Speed (triangle), Pseudoword Speed (asterisk).

Fig 5(A) and 5(B) plots the congruency effect by each of the four WIAT reading score metrics in the faces and words tasks respectively, and statistics are reported in Table 3. Considering first the faces task, for dyslexic participants greater holistic processing in the faces task is associated with better reading scores in both word and pseudoword accuracy and in word and pseudoword speed. This is not the case for the typical readers, where holistic processing in the faces task shows no obvious association with any of the reading metrics. Turning to the words task, greater holistic processing is associated with better reading scores in all four metrics for the dyslexic groups, whereas for the typical reader group greater holistic processing in the words task is associated with poorer performance in word and pseudoword accuracy but unrelated to speed. Respectful of encouragement to move away from the null-hypothesis significance testing framework [37] we plot estimated regression coefficients (slopes) with their 95% confident intervals using dot-and-whisker plots [38] in Fig 6. A clear pattern is evident whereby a higher congruency effect for dyslexic readers is predictive of better reading scores in all four metrics (word accuracy, pseudoword accuracy, word speed and pseudoword speed) and this is the case for both the faces and the words task. In contrast, for the typical readers, the congruency effect in the faces task is not predictive of reading scores while a higher congruency effect in the words task is predictive of lower word and pseudoword reading. Therefore, automatic and obligatory attention to all parts of a stimulus, as measured in the faces and words tasks, clearly relates to reading strategy as discussed below.

**Table 3:** Pearson’s r with associated p-value.

**Correlation with Congruency Effect for Faces**				
**Group**	**Word Accuracy**	**Pseudoword Accuracy**	**Word Speed**	**Pseudo-word Speed**
**Dyslexic**	.45 [.10, .70], *p* = .015	.39 [.03, .66], *p* = .037	.28 [-.09, .59], *p* = .139	.49[.15, .73], *p* = .007
**Control**	.09 [-.28, .45], *p* = .628	.09 [-.29, .44], *p* = .644	.05 [-.32, .41], *p* = .799	.18 [-.20, .51], *p* = .350
**Correlation with Congruency Effect for Words**				
**Group**	**Word Accuracy**	**Pseudoword Accuracy**	**Word Speed**	**Pseudo-word Speed**
**Dyslexic**	.30 [-.08, .60], *p* = .119	.27 [-.12, .58], *p* = .170	.39 [.02, .67], *p* = .038	.43[.07, .69], *p* = .023
**Control**	-.34 [-.63, .02], *p* = .064	-.41 [-.67, -.05], *p* = .028	.15 [-.23, .49], *p* = .428	.02 [-.35, .39], *p* = .909

*Note*. 95% CI are shown in brackets.

Further exploration using multiple regression on the dyslexia data only (performance on the faces task not being predictive of any reading metric for the typical readers) is summarized in Table 4. In the case of Word and Pseudoword Speed, the two different Congruency Effects (in the words task and in the faces task) acts as independently predictors. However for the Word and Pseudoword Accuracy score, the addition of the Congruency Effect in Words Task adds only marginal extra prediction (p<0.10).

**Table 4:** Multiple regression output.

** **	**Word Accuracy**			**Pseudoword Accuracy**		
***Predictors***	*Estimates*	*CI*	*p*	*Estimates*	*CI*	*p*
**(Intercept)**	50.77	43.18 – 58.36	<0.001	22.00	13.34 – 30.67	<0.001
**ceFaces**	43.43	9.23 – 77.62	0.015	44.71	5.66 – 83.77	0.027
**ceWords**	0.03	-0.00 – 0.07	0.081	0.03	-0.01 – 0.08	0.094
** **	**Word Speed**			**Pseudoword Speed**		
***Predictors***	*Estimates*	*CI*	*p*	*Estimates*	*CI*	*p*
**(Intercept)**	25.60	18.44 – 32.75	<0.001	15.94	11.41 – 20.47	<0.001
**ceFaces**	33.47	1.20 – 65.73	0.043	35.16	14.73 – 55.59	0.002
**ceWords**	0.04	0.00 – 0.07	0.035	0.03	0.01 – 0.05	0.009

*Note*. ceFaces–Congruency Effect in Faces Task, ceWords–Congruency Effect in Words Task.

## Discussion

We compared the performance of college students with dyslexia and age matched typical readers on two perceptual tasks, the Vanderbilt Holistic Face Processing Task (VHFPT) and the Holistic Word Processing Task (HWPT), that each yield a measure of holistic processing known as the ‘congruency effect’. This metric captures the extent to which participants automatically attend to information that is spatially nearby but irrelevant to the task at hand. In both the VHFPT and the HWPT, the extraneous or irrelevant information may benefit performance when it is congruent with the information that participants are asked to attend to, or may disadvantage performance when it is incongruent. The congruency effect is calculated as a difference score for performance on ‘congruent’ and ‘incongruent’ trials and serves as an index of holistic processing. Our results show, for the first time, that holistic processing of faces is comparable in dyslexic and typical readers but that dyslexic readers show greater holistic processing of words, at least for the specific tasks at hand. Furthermore, we show that these measures of holistic processing predict performance on a standardized reading task, the WIAT-3, with a more holistic style in *both* the faces and words task associated with better reading scores—specifically, more accurate and faster reading of both words and pseudowords—for dyslexic readers. In contrast, a more holistic style on the words task predicts less accurate reading of both words and pseudowords for typical readers.

Below we discuss how these findings compare to recent research on anomalous visual processing in developmental dyslexia and to a rapidly evolving literature on the role of visual attention in dyslexia. Finally, we consider how our finding of enhanced holistic processing in dyslexic readers–where holistic processing is defined in the strict sense of automatic attention to the whole stimuli–may guide our conceptualization of the visual deficit in dyslexia.

Starting with the faces task, many aspects of our findings (specifically, with the control participants) replicate directly those of [29] while also revealing interesting similarities between the dyslexic and control groups. Firstly, error rates are comparably high (~35%) to previous reports and are equal across dyslexic and typical readers. Second, accuracy is considerably higher on congruent than on incongruent trials as expected from [29] and this was the case for both dyslexic and typical readers. Although this was not an explicitly timed task, the response time data show that dyslexic participants are slower than typical readers to correctly match the target regions across the study face and the test faces. Yet this group difference was not modulated by congruency, with both dyslexic and typical readers showing comparable advantage on congruent trials.

This finding is consistent with recent reports of a general impairment in ventral stream processing in dyslexia that may lead to subtle differences in face processing but not to specific impairments in holistic processing [19, 20]. [20] found that typical readers showed an advantage over dyslexia readers on the Cambridge Face Memory Test, but that both groups were comparably impaired by stimulus inversion suggesting that there is no specific impairment in holistic processing in dyslexia. These authors also used the VHFPT and report that typical readers performed with higher accuracy (61.3%) than dyslexic readers (59.7%)–a result reported as ‘marginally significant’—but as this advantage was not specific to the congruent condition, they argue that this cannot be attributed to poorer holistic processing in dyslexic readers [20]. While noting that dyslexic and typical readers performed with comparable accuracy in our study, differently than in [20], our findings that the congruency effect is comparable between the groups strengthens previous conclusions that holistic processing of faces is not impaired in dyslexia. Research by [19] has been similarly motivated by the question of whether anomalous visual processing in dyslexia is specific to words or extends to other classes of visual objects. They report slower response times by dyslexic readers compared to typical readers in matching faces but not in matching cars, suggesting that visual impairments in dyslexia extend beyond words. However, as inverting the stimuli led to comparably slower performance in both groups there is no suggestion of a specific impairment in holistic processing.

Turning to the words task we note that aspects of our findings (specifically with the control group) map directly onto those of [25]. Participants are slower in the incongruent than in the congruent condition and this ‘congruency effect’ is greater for aligned than misaligned trials as reported by in their Study 1 [25]. With regard to group differences we find that, while dyslexic participants are slower than controls overall there is no difference in sensitivity between the groups. This is consistent with findings from our previous research [39] which reports that dyslexic participants are slower to respond than typical readers but show comparable sensitivity in a novel non-reading task that encompasses aspects of the ‘word superiority’ and ‘word inversion’ paradigms.

In the current study we find that dyslexic participants show a stronger ‘congruency effect’ than controls on the words task. Specifically, while both dyslexic and control participants were susceptible to interference from the unattended part of the stimuli, and more so when the two halves of words were properly aligned, dyslexics were more susceptible to this interference than controls. While it is difficult to directly compare with the findings of [25] is notable that in their Study 2—which compared the performance of native English speakers with those for whom English is their second language–the native English speakers showed a more marked ‘congruency effect’. This suggests that readers with more experience use more holistic processing than those with less experience. While all participants in the current study were college students—and reading is an integral part of college life–it would be difficult to argue that dyslexic students are the more expert readers. Interestingly, a recent paper by [40] shows that adults with dyslexia recognize Chinese characters with stronger holistic processing than controls.

Similarly, it is also difficult to directly compare the findings of the current study to those of [39] who utilized a very different task to compare the use of holistic processing between dyslexic and typical readers. As in the face perception tasks utilized by [20] and [19] stimulus inversion was used by [39] as a way to explore holistic processing of words. Specifically, participants were asked whether pairs of words (which were identical or varied by one letter, and which were intact or jumbled) were the same or different and word pairs were presented in both upright and inverted orientation. [39] show a more marked inversion effect for control than for dyslexic participants. Specifically, for short 4-letter words, response times to discriminate inverted stimuli was comparable across the two groups whereas for upright stimuli dyslexics were markedly slower than the typical readers suggesting that they benefit less from holistic cues. Although both groups showed clear evidence of holistic processing in that study, typical readers showed more marked holistic processing than dyslexic readers. In contrast, in the current study the dyslexic participants show a definite congruency effect that is an accepted marker of holistic processing *and* a more marked effect than their peers in the typical reader group. It may be that typical readers have more flexibility in how they perform word processing tasks and can switch more easily between holistic and analytic processing as required.

A central finding of this research is that the congruency effect, as measured in both the faces task and in the words task, is predictive of dyslexic participants’ reading scores with more holistic processing in both tasks associated with higher accuracy in reading words and pseudowords and in faster reading of words and pseudowords. This is evident in Fig 6 where we plot estimated regression coefficients (slopes) with their 95% confident intervals. Across both tasks, the obligatory attention to extraneous information captured by the congruency effect is predictive of better—faster, more accurate—reading in dyslexic readers. In contrast, holistic processing in the faces tasks is not predictive of reading performance in the control group, and holistic processing in the words task is only predictive of reading accuracy and that association runs counter to the pattern seen for the dyslexic readers. For the typical readers more holistic processing in the words task is associated with less accurate word and pseudoword reading.

Interestingly, a recent study [41] that a higher congruency effect for words is associated with more efficient performance of *typical readers* in a lexical decision task. In that study, participants were asked to indicate as quickly as possible whether a presented letter string was a real word or not, the stimuli consisting of 4, 5 and 6-letter words (and associated pseudowords) of both low and high frequency. The negative correlation between the magnitude of the word-frequency effect (where a smaller word frequency effect is associated with more efficient word processing) and the word congruency effect means that the more efficient readers were less able to selectively ignore extraneous information in the word composite task. This finding appears to run counter to the present finding that a higher congruency effect on the word task is associated with less accurate reading of words and pseudowords. Obviously, the two tasks–reading words and pseudowords aloud in the current study and making a speeded lexical decision task in- are quite different, the reading aloud task requiring sub-lexical phonological processes. It would be interesting to see whether the current findings on the relationship between the congruency effect for faces/words and performance on the reading tasks reported for dyslexic readers holds also for lexical decision tasks.

In response to a reviewer’s comments we note that the tasks we used to measure holistic processing of words and faces differ in a number of ways. For example, the VHFPT utilises a 3AFC task in which participants are asked to attend to one specific target region of a study face and to subsequently locate the matching identity in the same target region from 3 test faces. In contrast, the HWPT or word composite task uses a same-different paradigm in which a study word is presented and participants are subsequently cued to which side of the test word they should attend to in deciding whether it is the same or different than the corresponding half of the study word. However, common to both tasks is the requirement that participants *selectively attend* to one part of a complex stimulus while ignoring a spatially adjacent part of the stimulus, and the congruency effect is a measure of their ability or inability to ignore this extraneous information. We stress that we are not claiming that ‘holistic processing’ (as a perceptual style) is the same for faces and for words, but simply that the congruency effects serve as a measure of the involuntary tendency to integrate information across the stimulus in these different tasks.

Before considering the implications of these findings, we also draw attention to the fact that, while all participants in the current study reported normal or corrected-to-normal vision, it is notable that those with dyslexia (22/30) show a greater incidence of refractive errors and other issues with vision than those without dyslexia (11/29). Myopia or short-sightedness is marked in the dyslexic (17/30) compared to the typical reader (5/29) sample. Reduced visual acuity has been previously reported as being significantly associated with dyslexia [42] but others report no association between refractive error and dyslexia [43]. It is also possible that the typical readers in this study had unusually low rates of myopia, as national statistics show prevalence rates of ~19% in children aged 12–13 years which would be expected to be higher in college aged young adults [44].

By way of general conclusions, our results join others in showing subtle impairments in high level visual processing, including in memory for faces, perceptual matching of faces, within-category discrimination of other objects [20, 21] and in recognition and matching of words and faces [19]. Collectively, these findings suggest that visual deficits underlying dyslexia are more ‘domain general’ than ‘domain specific’ in that they affect the recognition of objects other than words [19]. Interestingly, similar findings have been reported in cases of alexia, an acquired impairment in reading following brain injury and historically also referred to ‘letter-by-letter reading’, ‘word blindness’, ‘word form dyslexia’ and ‘acquired dyslexia’ [45]. For example, [46] describe four patients with pure alexia, arising for unilateral damage to left inferior occipitotemporal lobe, who show poorer performance on a face matching task than controls. Similar to the results now emerging in research on developmental dyslexia, these impairments in face processing in alexic patients are described as ‘mild’. Interestingly, brain imaging research points to a common dysfunction in left occipitotemporal cortex (the visual word form area) in both acquired and developmental dyslexia [47, 48].

A second conclusion is that the visual deficit in dyslexia has a strong attentional component, and we base this observation on our findings that holistic processing of both words and faces strongly predicts word and pseudo word accuracy and speed in dyslexic readers. In contrast, holistic processing of faces is unrelated to reading scores in typical readers, and where holistic processing of words is related to reading accuracy, the predictions run counter to those for the dyslexic group. While ‘holistic processing’ is an elusive concept in both definition and measurement [49], the tasks we use in this study operationalize holistic processing in terms of selective attention. Variously described as measuring obligatory attention to all parts of an object or, analogously, as a failure of selective attention to parts of an object [25, 50] this form of perceptual processing is traditionally associated with expertise, see [51, 52] for debate.

In a comprehensive review of accounts of dyslexia, [13] note the heterogeneity of the dyslexic population and present evidence that anomalous attentional processing may be the core deficit in a subset of dyslexic children. Since then, the independence of deficits in phonological processing and in visual attention disorders as contributing factors to dyslexia has been demonstrated in both French and English speaking samples [14]. These studies, notable for their use of larger sample sizes that are necessary to explore heterogeneity in the disorder, join others emphasising the role of visual factors in dyslexia. For example, using cluster analysis with a sample of 316 Italian children [12] show distinct groupings, both of whom show impairment in visual tasks but only one of whom shows phonological impairment. The authors conclude that visual impairment is central to dyslexia which cannot be explained with reference to a primary phonological impairment. And specific to adult readers, [53] report that college students with dyslexia show poorer performance than their peers in tasks involving visual discrimination of novel grid-like patterns and in visuospatial working memory tasks which are known to require attentional control.

How might these findings inform the interpretation of the results from our current study? The use of visuo-spatial tasks with reports of anomalous attentional factors provide a common theme to these diverse studies. In the current research, both the VHFP and the HWPT may be conceptualized as tasks of selective attention with dyslexic participants showing a comparable tendency toward holistic processing in the faces task and a greater tendency toward holistic processing in the words task. Furthermore, while the congruency effect on the faces task and on the words task are both predictive of reading scores for the dyslexic group with a more holistic style associated with improved reading, the association between holistic processing and reading performance for the control group is only seen in the case of the words tasks where a more holistic style is associated with poorer accuracy in word and pseudoword reading. It is important to consider these very different patterns of association for the dyslexic and typical reader groups in light of their very different performance on the WIAT reading tests (Fig 1, Table 1); these groups differ substantially in their reading performance with typical readers attaining significantly higher levels of accuracy on average.

Reading involves the analysis of visual word forms at different spatial scales, including noting letter combinations at both coarse and fine scales that signal spelling regularities [9] and this combined use of global and analytic processing is central to attention-focused models of reading [13]. Particularly in languages with opaque orthographies such as English, we suggest that efficiency or fluency in reading may be associated with the ability to switch strategy as needed, rather than with an exclusively holistic strategy. This consideration is likely relevant to understanding the broader question of what underlies reports of ‘mild’ impairments in non-reading tasks in dyslexia that hint to differences in ventral stream processing underlying ‘perception expertise’, e.g., [19, 20]. As noted by [51] might more usefully be conceptualised in terms of attentional as well as purely perceptual factors.

## Conclusions

We replicate recent findings that dyslexic readers show mild impairment in visual, non-reading tasks including in a face perception and a word perception task that both yield a metric of holistic processing. Further we show that this metric, the ‘congruency effect’, predicts reading performance in dyslexic readers with a more holistic style associated with better accuracy and speed scores. In contrast, a more holistic style on the words task is associated with poorer word and pseudoword accuracy scores in typical readers. This suggest that selective attention plays a different role in the reading strategies of dyslexic and typical readers.

## Supporting information

S1 Fig The upper panel depicts each of the nine possible target regions in the red segments. The lower panel contains an example of a congruent (upper row) and an incongruent trial (lower row) using the top half target region which is highlighted in red. On the congruent trial, the target segment in the study image and in the (correct response) test image are two different images of the same person (Person A) *and* the non-target region (the bottom half of the face) are also images of the same person (Person B). On the incongruent trial, the target segment in the study image and in the (correct response) test image are two different images of the same person (Person A) *but* the non-target regions are images of different people. Target segments are outlined in colour for illustration purposes only and were not used in the actual experiment.
(TIF)

S2 Fig (A) Examples of four-letter study and test words in congruent and incongruent conditions where the first two letters of the study and test are the same or different. (B) The temporal sequence of the stimuli presented. Based on Wong et al. (2011).
(TIF)
