# Supplementary figures and images for: Holistic processing of faces and words predicts reading accuracy and speed in dyslexic readers
Source: PLoS One. 2021 Dec 15;16(12):e0259986. doi: 10.1371/journal.pone.0259986 (PMC8673614; doi:10.1371/journal.pone.0259986)

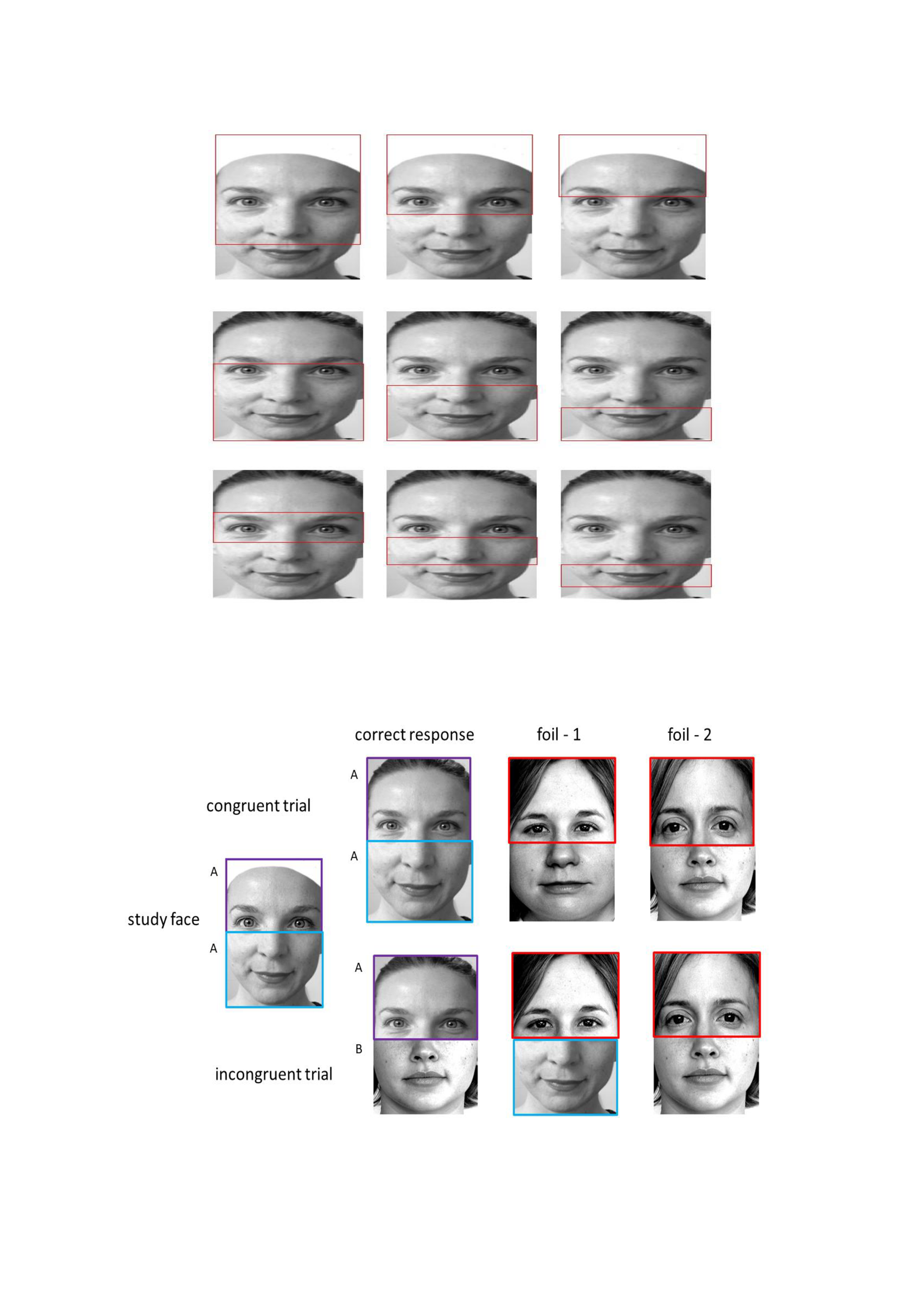

Supplement: S1 Fig — The lower panel contains an example of a congruent (upper row) and an incongruent trial (lower row) using the top half target region which is highlighted in red. On the congruent trial, the target segment in the study image and in the (correct response) test image are two different images of the same person (Person A) and the non-target region (the bottom half of the face) are also images of the same person (Person B). On the incongruent trial, the target segment in the study image and in the (correct response) test image are two different images of the same person (Person A) but the non-target regions are images of different people. Target segments are outlined in colour for illustration purposes only and were not used in the actual experiment. (TIF) [file pone.0259986.s001.tif]

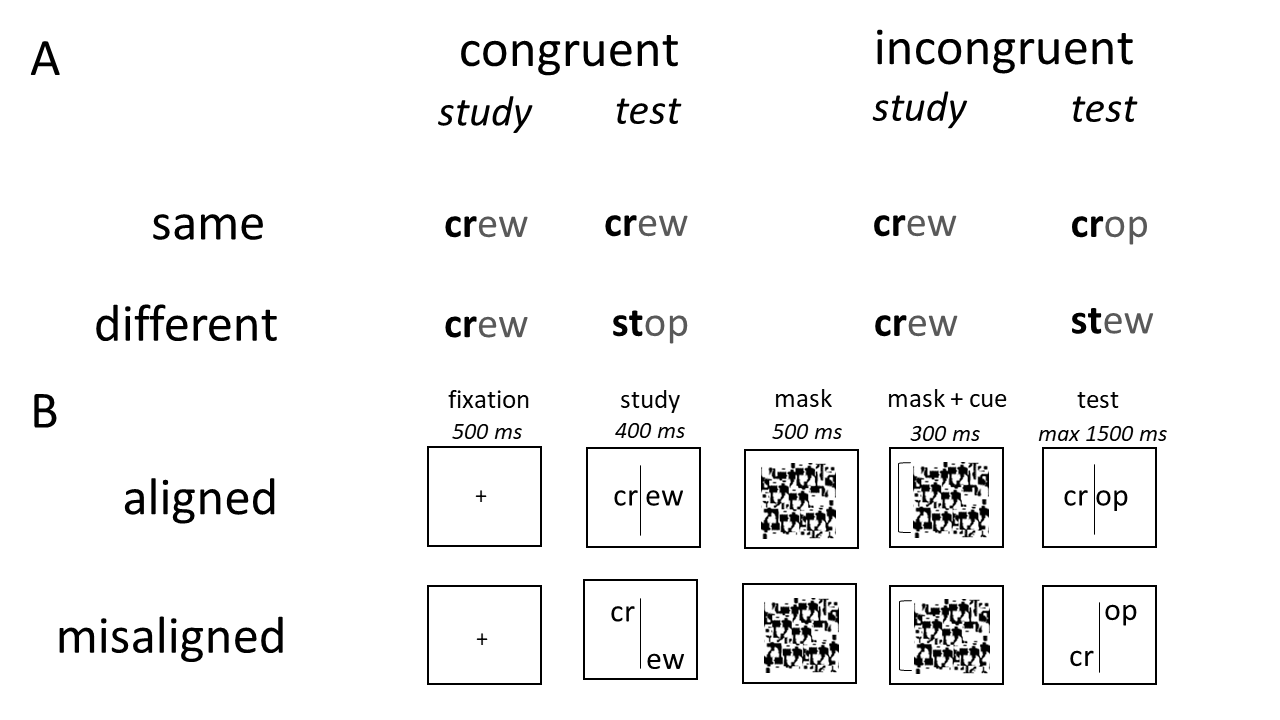

Supplement: S2 Fig — (A) Examples of four-letter study and test words in congruent and incongruent conditions where the first two letters of the study and test are the same or different. (B) The temporal sequence of the stimuli presented. Based on Wong et al. (2011). (TIF) [file pone.0259986.s002.tif]
